# Bis(trifluoromethylsulfonyl)imide Ionic Liquids Applied for Fine-Tuning the Cure Characteristics and Performance of Natural Rubber Composites

**DOI:** 10.3390/ijms22073678

**Published:** 2021-04-01

**Authors:** Anna Sowińska, Magdalena Maciejewska, Anna Grajewska

**Affiliations:** Institute of Polymer and Dye Technology, Lodz University of Technology, Stefanowskiego Street 12/16, 90-924 Lodz, Poland; anna.grajewska@edu.p.lodz.pl

**Keywords:** ionic liquids, bis(trifluoromethylsulfonyl)imides, natural rubber, composites, cure characteristics, mechanical properties, thermo-oxidation

## Abstract

The goal of this work was to apply ionic liquids (ILs) with bis(trifluoromethylsulfonyl)imide anion (TFSI) for fine-tuning the cure characteristics and physico-chemical properties of elastomer composites based on a biodegradable natural rubber (NR) matrix. ILs with TFSI anion and different cations, such as alkylpyrrolidinium, alkylammonium, and alkylsulfonium cations, were applied to increase the efficiency of sulfur vulcanization and to improve the performance of NR composites. Thus, the influence of ILs on the vulcanization of NR compounds, as well as crosslink density and physical properties of NR vulcanizates, including tensile properties, thermal stability, and resistance to thermo-oxidative aging was explored. The activity of ILs seems to be strongly dependent on their cation. Pyrrolidinium and ammonium ILs effectively supported the vulcanization, reducing the optimal vulcanization time and temperature of NR compounds and increasing the crosslink density of the vulcanizates. Consequently, vulcanizates with these ILs exhibited higher tensile strength than the benchmark without IL. On the other hand, sulfonium ILs reduced the torque increment owing to the lower crosslinking degree of elastomer but significantly improved the resistance of NR composites to thermo-oxidation. Thus, TFSI ILs can be used to align the curing behavior and performance of NR composites for particular applications.

## 1. Introduction

**Scheme 1:**
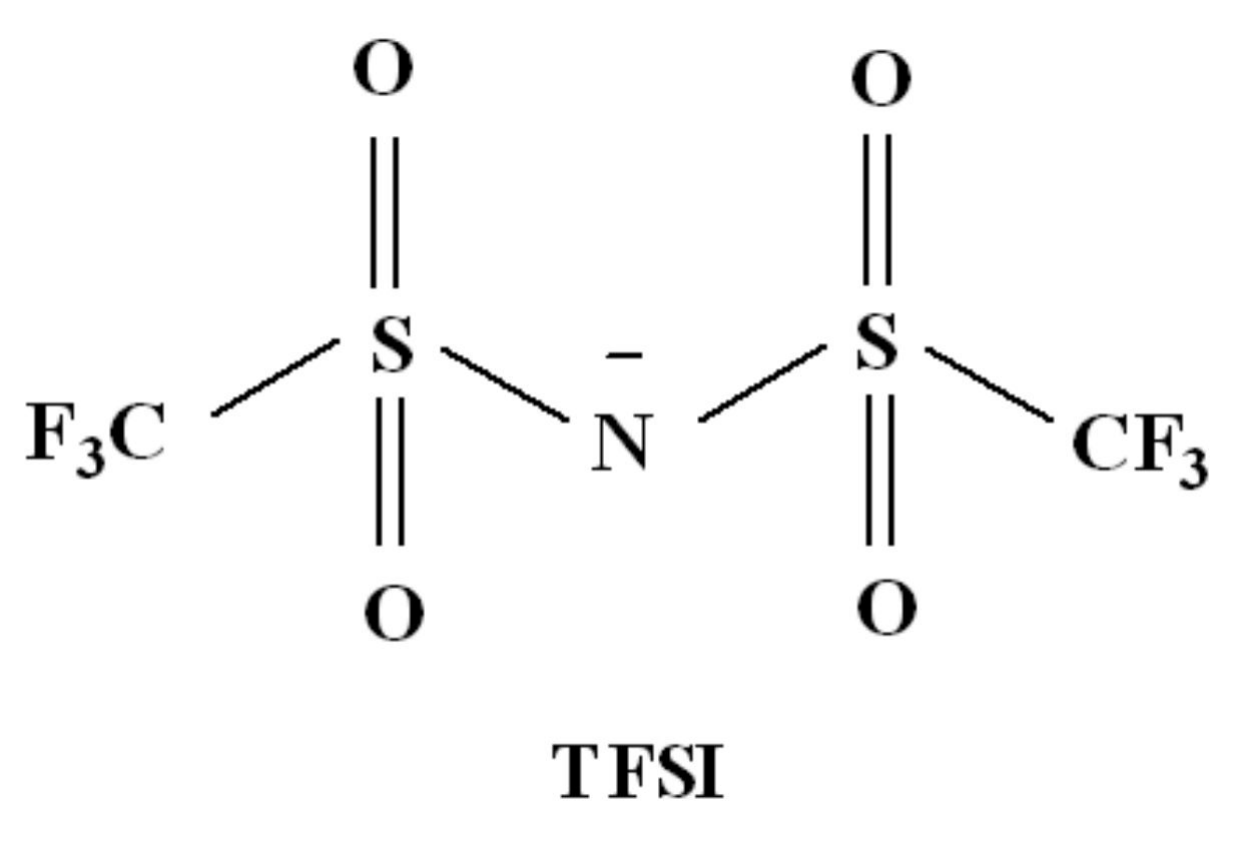
Structural formula of bis(trifluoromethylsulfonyl)imide (TFSI) anion.

Ionic liquids (ILs) provide a class of solvents composed only of ions [1]. They consist of an organic asymmetric cation and an anion which can be both organic and inorganic. Typical ILs contain large organic cations, such as imidazolium, ammonium, pyridinium, piperidinium, or pyrrolidinium cations, as well as halogen, fluorinated, or organic anions [1,2,3]. These salts are characterized with many useful and unique properties in comparison with traditional solvents [4,5,6] and are often referred to as environmentally-friendly and safe solvents because they have high thermal stability and essentially zero vapor pressure at normal temperatures. The most popular and often used IL systems consist of heterocyclic cations such as imidazolium, which is often paired with halided or fluorinated anions such as tetrafluoroborate (BF_4_^−^), hexafluorophosphate (PF_6_^−^), or bis(trifluoromethylsulfonyl)imide (Tf_2_N^−^, TFSI) [3,7,8]. Among different types of ILs, bis(trifluoromethylsulfonyl)imides have been reported to be convenient for various practical applications [9,10,11]. The weakly coordinating TFSI anion has a remarkable effect on the solvent abilities of the ILs. In the case of the hydrogen-bonding solutes, ILs with the TFSI anion generally exhibit substantially lower solubility than salts with other anions. Two specific properties of this anion restrict its interaction abilities. First, the specific interactions between the neighboring sulfur and nitrogen atoms (Scheme 1) in the TFSI anion delocalize the negative charge mainly along the anion′s S-N-S moiety, while only a small portion of the charge remains delocalized on the sulfonyl oxygen atoms. This way, the oxygen atoms and terminal -CF_3_ groups shield the negative charge and restrict Coulomb interactions with the coupled cation, while increasing the ion mobility [12,13]. However, the ability of the sulfonate oxygens to accept protons must be decreased as well, being reflected in much lower hydrogen bond basicity of the anion. Steric hindrance is mainly a consequence of S-N-S angle (125°) slightly larger than that in the trigonal pyramidal molecular structure (∽107°) and bulkiness of the -SO_2_CF_3_ groups in the anion, which shield the negative charge. This hindrance sustains the aforesaid charge delocalization and helps in reducing the interaction ability of the TFSI anion. These two aspects may cause that ILs with the TFSI anion are not miscible with water. In addition, the TFSI anion significantly lowers the melting points of ILs. It results from delocalization of the negative charge in the TFSI anion, that spread from the central nitrogen atom to adjacent atoms of sulfur and to a lesser extent to four oxygen atoms bonded to sulfur atoms [13,14]. The charge delocalization effect was also reported by Kurig et al. [15] and Shaplov et al. [16] Thus, ILs with TFSI anion are usually liquid in the temperatures above 0 °C. Generally, the viscosity of most ILs with various anions is higher than that of conventional organic solvents. This could be a disadvantage in some industrial applications of ILs, for example, those involving mass transfer processes or requiring stirring operations. ILs with TFSI anion, especially those with pyrrolidinium or sulfonium cations, show significantly lower viscosities than ILs with other (e.g., morpholinium) cations [17]. Regardless of the structure, ILs are characterized with high decomposition temperatures [18] and inherently high normal boiling temperatures [19]. It is worth noting that the thermal decomposition temperature of some ILs reaches even approximately 400 °C and depends mainly on the anion type, the influence of cation is less significant. For the same cation, the thermal stability of ILs decreases as follows: [Tf_2_N]^−^ > [PF_6_]^−^ > [BF_4_]^−^ > halides [20]. The change in the length of alkyl chain in the same type of cation, such as ammonium, imidazolium, or pyridinium, has little influence on the ILs decomposition temperature [21].

As was already mentioned, incorporation of TFSI anion into ionic liquid molecule results in rather low viscosity and good electrochemical stability compared to ILs with other anions. As a consequence, ILs with TFSI anion can be successfully used as electrochemical double-layer capacitors (EDLCs) [22,23]. One of the most widely used in EDLCs is N-butyl-N-methyl pyrrolidinium bis(trifluoromethylsulfonyl)imide [24]. Its disadvantages are rather high viscosity (as compared to TFSI-based ILs with other cations) and comparatively poor ionic conductivity. Consequently, EDLC based on this ionic liquid requires elevated temperatures to work properly. A good alternative for this ionic liquid to be applied as electrolytes are sulfonium ILs with TFSI anion. Most of ILs containing trialkylsulfonium cation, e.g., diethylmethylsulfonium bis(trifluoromethylsulfonyl)imide, exhibit comparatively low viscosity and higher ionic conductivities as compared to pyrrolidinium ILs [25].

Sulfonium ILs with TFSI anion such as triethylsulfonium bis(trifluoromethylsulfonyl)imide can also be used as corrosion inhibitors for cleaning some parts of desalination plants. They reduce the rate of corrosion by the adsorption process [26].

Regarding ammonium ILs with TFSI anion, similarly to those with pyrrolidinium or sulfonium cations, these compounds were reported to be used mainly as electrolyte components for high-energy electrochemical storage devices [27,28,29].

Applications of ILs with TFSI anion and pyrrolidinium, ammonium, or sulfonium cations in the elastomer composites, especially based on the natural rubber, are not common and have not been reported in the literature so far. However, good thermal, electrochemical, and chemical stability over a wide temperature range cause these ILs to be successfully used in elastomer composites not only as conductive additives but also for fine-tuning the cure characteristics or performance of elastomers.

Natural rubber (cis-1,4-polyisoprene) is obtained from the rubber tree (*Hevea brasiliensis*), thus it is an inexpensive, renewable, non-toxic polymer and creates fewer health hazards [30,31] as compared to rubber goods made of synthetic rubbers. Owing to its low price and functional properties, it is the most used elastomer in industry worldwide and has a wide range of possible applications in different sectors of economy, such as automotive, construction, footwear, and furniture industries [32]. Natural rubber (NR) elastomer exhibits a high degree of deformation at low stresses in the range of operating temperatures. It is nonpolar and therefore has relatively good resistance to polar solvents. Moreover, compounds based on cis-1,4-polyisoprene are characterized with good extrudability and calenderability, and can be easily and quickly vulcanized using a conventional sulfur curing system. NR vulcanizates are characterized with high tensile strength due to the stretch induced crystallization [33]. On the other hand, NR has double bonds in its structure, so it is highly unsaturated. This results in poor resistance of NR to oxidation and consequently precludes long-term exploitation of rubber products due to their susceptibility to accelerated aging processes [32,34]. Therefore, to limit the negative effect of aging and degradation processes on the properties of NR vulcanizates, it is necessary to use various types of stabilizers and anti-aging agents. The proper curing system and fillers are other crucial ingredients to obtain the desired properties of the NR vulcanizates. The most significant is to develop the composition of NR compounds which enables to produce materials that are environmentally friendly, safe during processing, cost competitive, and applicable.

Most of publications related to NR concern the use of different fillers, such as carbon black (CB) and conductive carbon black (CCB) or carbon nanotubes (CNTs) to improve mechanical performance and electrical conductivity of the elastomer composites. For example, Thaptong et al. investigated the effect of hybrid fillers based on CB as a primary filler and CCB or CNTs as secondary fillers on the cure characteristics and mechanical properties of NR composites [35]. Addition of CCB or CNTs reduced the scorch time and optimal vulcanization time of rubber compounds. Moreover, CNTs enhanced the hardness of vulcanizates by more than 10 ShA, which is a serious disadvantage in some applications of rubber products. Authors did not study the influence of fillers tested on the crosslink density, thermal stability, and resistance of the vulcanizates to aging, although these properties are also important from technological viewpoint. Fu et al. reported that application of carbon black N330 allowed to obtain NR vulcanizates with tensile strength higher than 20 MPa [36]. However, this required 40 phr of CB which made it difficult to homogeneously disperse this amount of filler. To achieve this goal, polyethylene glycol was grafted onto the surface of CB. Grafting was performed by chemical modification of CB using different chemicals, such as nitric acid, thionyl chloride, toluene, dibutyl dilaurate, and complicated procedure. On the other hand, ILs were reported to improve the dispersion of carbon fillers such as CB or CNTs in the elastomer matrix without necessity of fillers modification [37,38]. The influence of the grafted CB on the cure characteristics or the aging resistance of vulcanizates has not been discussed. Since grafted CB influenced the crosslink density, it is expected to influence the course of vulcanization as well as the aging behavior of the vulcanizates.

Studies on the applications of ILs in NR composites focus mainly on the alkylimidazolium salts with different anions [37,39,40,41]. ILs have been used to improve the dispersion of fillers and consequently, mechanical properties and/or conductivity of NR composites [37,38,39,40], as well as to produce the NR-based ionogels characterized with high ionic conductivity [41]. Most of the published studies concern the influence of ILs on the reinforcing effect of fillers, rheological properties, and conductivity of NR composites, ignoring the curing characteristics, thermal stability, or aging resistance of the vulcanizates, which is one of the most important weakness of this rubber, especially for outdoor use. To our knowledge, there is also no systematic research on the influence of the ILs structure on the crosslinking process and the functional properties of NR composites, although it can be expected that ILs, depending on their structure, may be successfully used to align the performance of elastomer composites for particular applications.

Thus, in this work, we applied various ILs with TFSI anion for fine-tuning the cure characteristics and physico-chemical properties of biodegradable elastomer composites based on the NR matrix. ILs with TFSI anion and alkylpyrrolidinium, alkylammonium, or alkylsulfonium cations were applied to support the sulfur vulcanization and to improve the performance of NR composites including their tensile properties and resistance to thermo-oxidation.

## 2. Materials and Methods

### 2.1. Materials

**Scheme 2:**
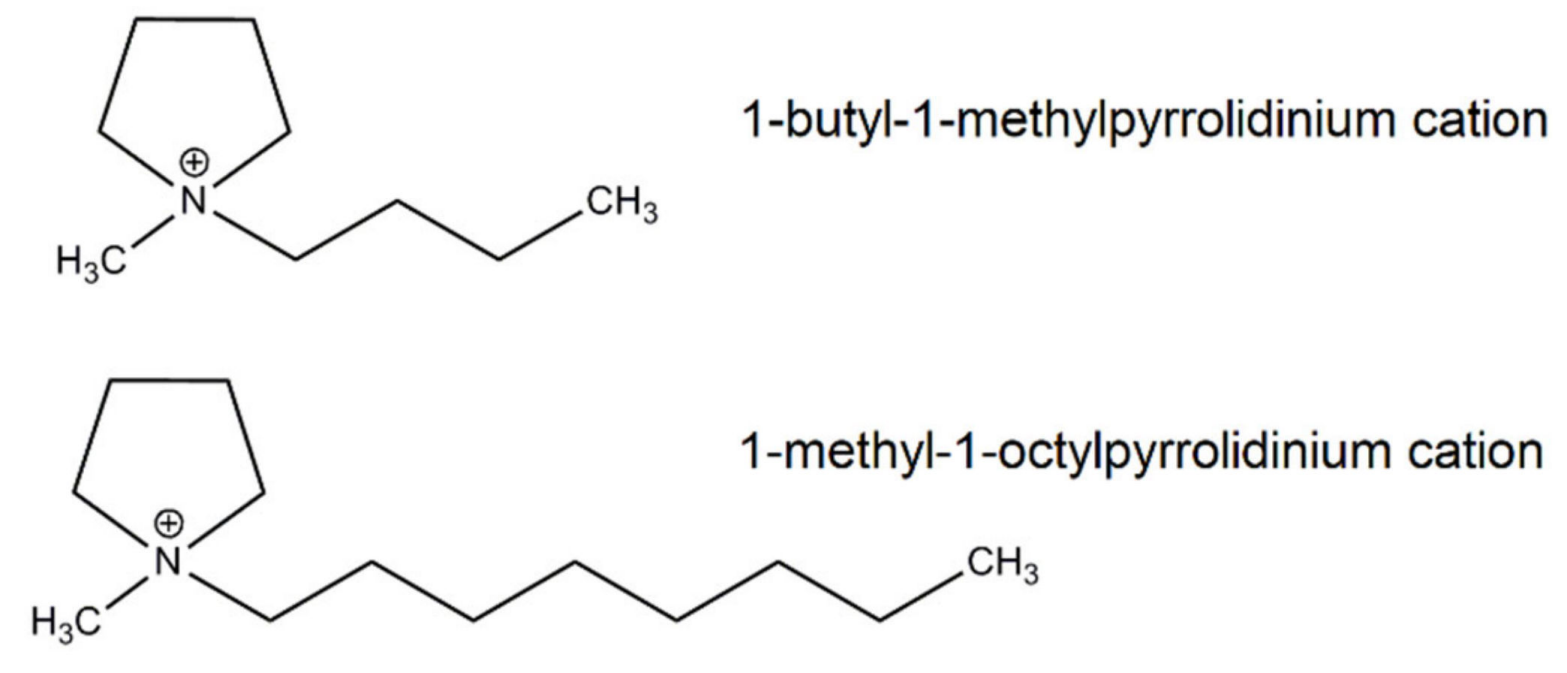
Structure of alkylpyrrolidinium cations of ILs.

**Scheme 3:**
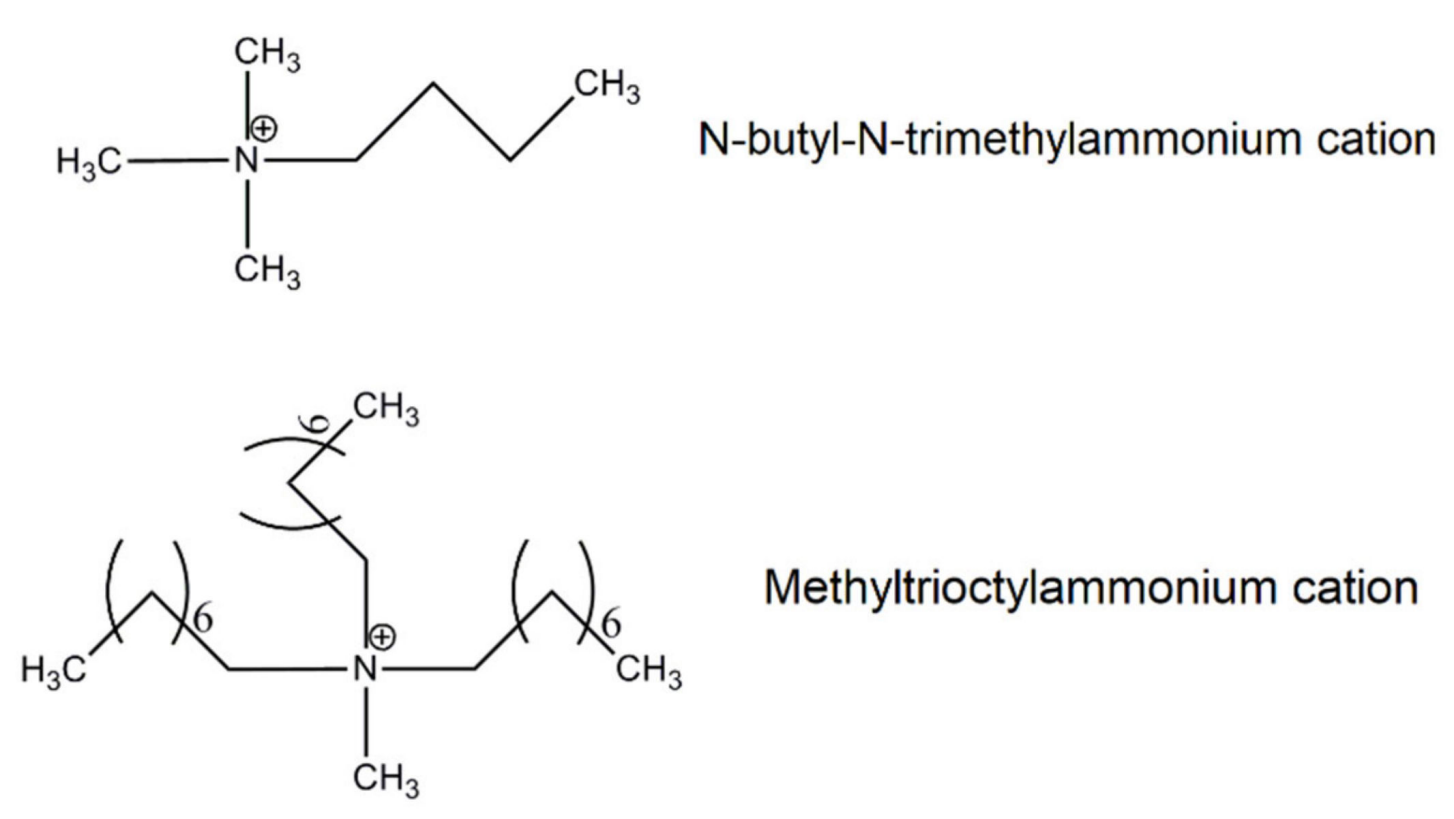
Structure of alkylammonium cations of ILs.

**Scheme 4:**
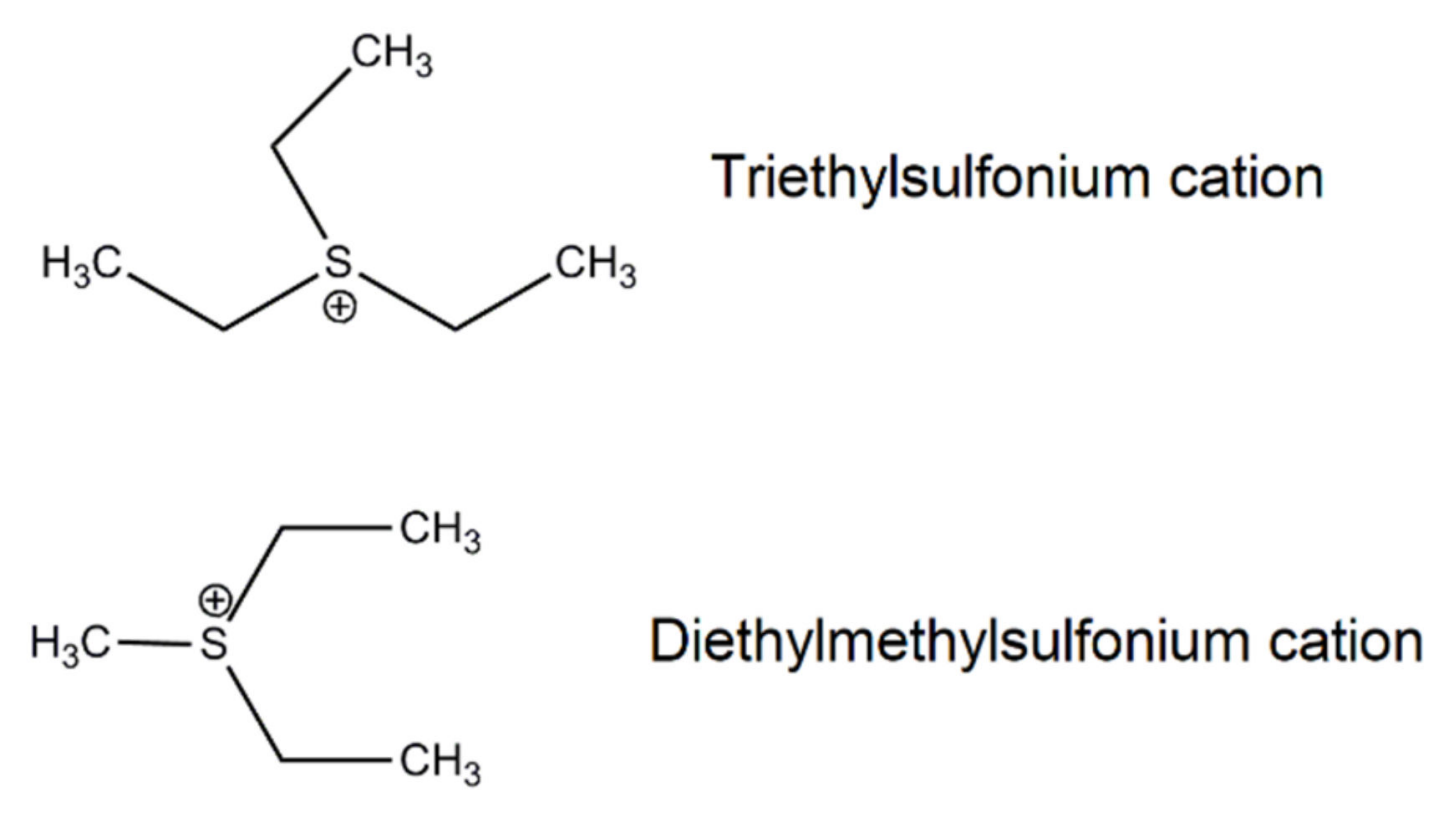
Structure of alkylsulfonium cations of ILs.

Natural rubber (NR, RSS1 type *cis*-1,4-polyisoprene) with a density of 0.930–0.988 g/cm^3^ was obtained from Torimex Chemicals, Lodz, Poland. Conventional curing system was applied, containing sulfur as a curing agent and 2-mercaptobenzothiazole (MBT) as vulcanization accelerator. These ingredients were supplied by Torimex Chemicals, Lodz, Poland. To activate the vulcanization, microsized zinc oxide with a specific surface area of 10 m^2^/g (ZnO) (Huta Będzin, Będzin, Poland) was mixed with ionic liquids (ILs) collected in Table 1 and then employed as vulcanization activator. The structures of ILs cations are presented in Scheme 2, Scheme 3 and Scheme 4. Carbon black (CB) N550, with a specific surface area of 40 m^2^/g and pH in the range of 7–10, was used as a filler (Konimpex, Konin, Poland). Additionally, stearin manufactured by Akzo Nobel (Amsterdam, The Netherlands) was used as a softener and dispersing agent.

### 2.2. Preparation and Characterization of NR Compounds

NR compounds were prepared using a rolling mill in a two-step procedure. First, the masterbatch was prepared, which consisted of the filler (CB), curing agent (sulfur), vulcanization accelerator (MBT), and stearin. The masterbatch was weighed and then cut into seven equal parts. Next, the activator such as ZnO previously mixed with a particular ionic liquid, was added to each of these pieces (with except of the reference rubber compound) and mixed up for additional 8 min. The general recipes of NR composites containing ILs are listed in Table 2 in parts per hundred of rubber (phr). A mass was the unit of measurement used in preparing the compositions of rubber compounds. The reference rubber compound contained ZnO without the ionic liquid.

NR compounds were cured at 160 °C, at 15 MPa pressure using the optimal vulcanization times determined during rheometric tests. The cure characteristics of NR compounds were investigated at 160 °C following the procedure described in ISO 6502 [42] using a rotorless curemeter MDR 2000 (Alpha Technologies, Hudson, Ohio, USA). The optimal vulcanization time (t_90_) was determined as the time for rheometric torque to reach 90% of the maximum achievable torque value, given by Equation (1), where *ΔS* is the torque increase during vulcanization, calculated as the difference between the maximum (*S_max_*) and minimum torque (*S_min_*). Using a similar equation, the scorch time (t_02_) was established:

(1) $$S_{90}=0.9\Delta S+S_{min}.$$

For studying the temperatures and the enthalpy of NR curing reactions, a differential scanning calorimeter DSC1 equipped with a STAR^e^ software (Mettler Toledo, Greifensee, Switzerland) was employed. The DSC curves were registered in the temperature range of −100–250 °C, with a heating rate of 10 °C/min. Nitrogen (80 mL/min) was used as the protective gas, whereas liquid nitrogen was applied to cool the sample before the measurement. The measurements were carried out for a small pieces of rubber compounds with a mass of approximately 9 mg, which were placed in a hermetically sealed aluminum crucible. The onset temperature of the peak corresponding to curing reactions was determined following the ISO 11357-1 [43] standard using the STAR^e^ software. According to the ISO 11357-1, the extrapolated onset temperature is the designated intersection point of the extrapolated baseline and the inflectional tangent at the beginning of the peak. The baseline and the inflectional tangent were determined from the temperature-dependent heat flow signal.

The crosslink density of NR vulcanizates was calculated based on solvent-swelling measurements according to the standard ISO 1817 [44]. Toluene was used as a solvent. The Flory–Rehner equation [45] was used to calculate the crosslink density with the Huggins parameter of elastomer-solvent (NR-toluene) interaction given by Equation (2), where *V_r_* is the volume fraction of elastomer in swollen gel [46]:

(2) $$\chi =0.478+0.228V_{r}.$$

The mechanical properties were examined by the universal testing machine Zwick Roell 1435 (Ulm, Germany) according to ISO 37 [47] standard procedures to study the tensile strength (TS) and elongation at break (EB) of NR vulcanizates.

The hardness of disc-shaped samples was examined using Shore′s method according to the standard ISO 868 [48] by Zwick Roell 3105 (Ulm, Germany) hardness tester.

The thermo-oxidative degradation of NR vulcanizates was conducted according to the ISO 188 standard [49]. Vulcanizate plates were stored in a drying chamber (Binder, Tuttlingen, Germany) at a temperature of 70 °C for 240 h. To estimate the resistance of the vulcanizates to thermo-oxidation, their mechanical properties and hardness after aging were examined and compared with the values characteristic for non-aged vulcanizates. To quantify the resistance of a material to thermo-oxidation, the aging coefficient *(AF)* was calculated according to Equation (3) [50,51], where TS is the tensile strength of vulcanizates and EB is the elongation at break:

(3) $$AF=\frac{{\left(EB\times TS\right)}_{after\;aging}}{{\left(EB\times TS\right)}_{before\;aging}}.$$

Thermal stability of pure ILs and NR vulcanizates was explored using thermogravimetric analysis (TG) by TGA/DSC1 (Mettler Toledo, Greifensee, Switzerland) analyzer following a two-step procedure. First, vulcanizates were heated in the temperature range of 25–600 °C in an argon atmosphere to study the pyrolysis of elastomer. Next, the gas was changed into air and heating was continued up to 900 °C. The heating rate during measurements was 20 °C/min, and the flow rates of argon and air were 40 mL/min.

## 3. Results and Discussion

### 3.1. Thermal Stability of Applied Ionic Liquids with TFSI Anion

**Figure 1:**
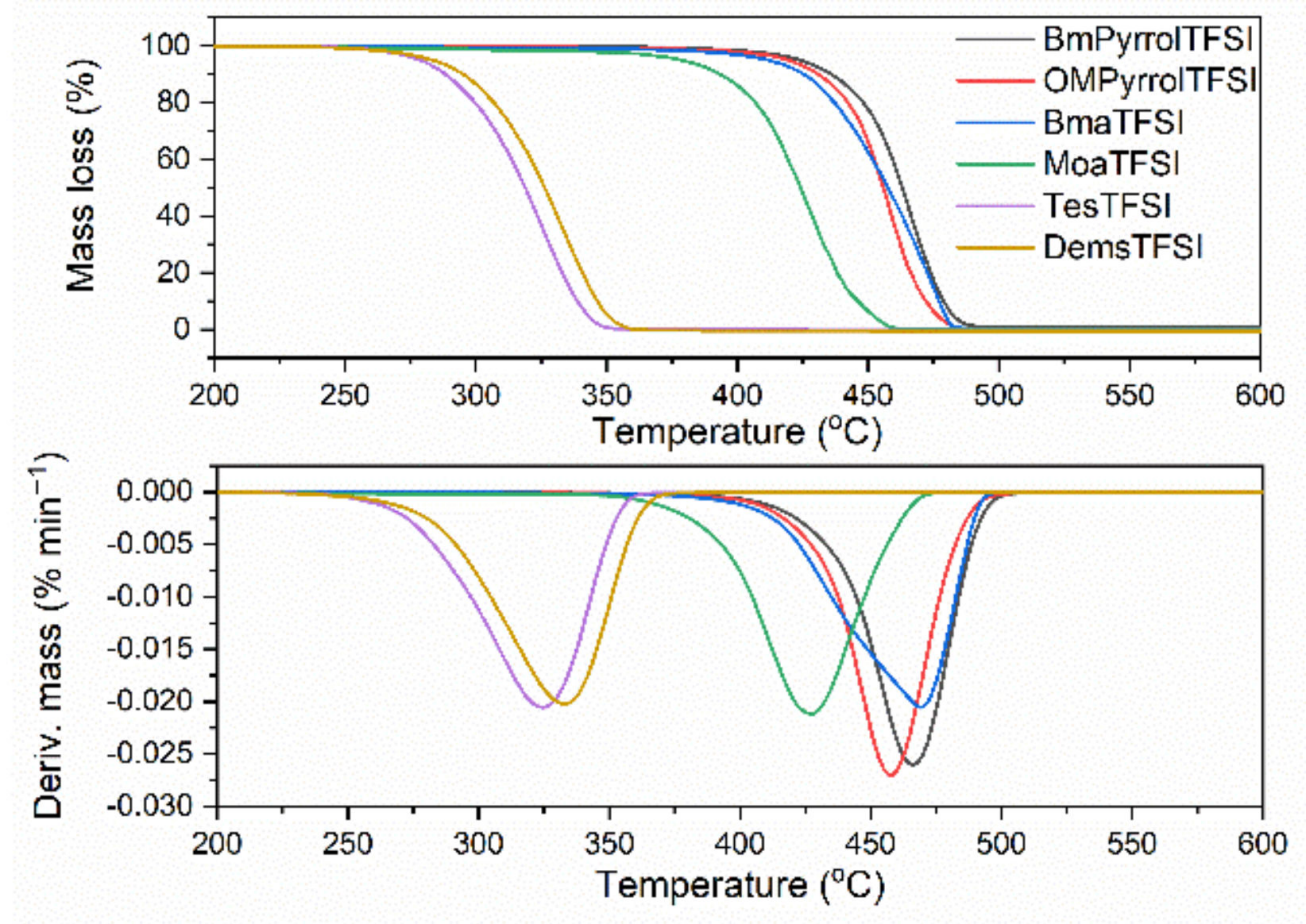
Thermogravimetric (TG) and derivative thermogravimetric (DTG) curves for ILs with TFSI anion.

The thermal stability of ionic liquids (ILs) is commonly known to depend on their structure, so the type of cation and anion. The studied ILs consist of the same bis(trifluoromethylsulfonyl)imide (TFSI) anion, but possess different cations such as alkylpyrrolidinium, alkylammonium, and alkylsulfonium, with different length of alkyl chains. Thus, they can be expected to show different thermal stability. Thermogravimetry (TG) was employed to establish the decomposition temperature of ILs at 5% of the mass change (T_5%_), which corresponds to the onset degradation temperature. Additionally, the DTG peak temperature (T_DTG_) was determined as the temperature of main decomposition step. The results are presented in Figure 1 and Table 3.

Figure 1 shows the course of the TG (thermogravimetric) and DTG (derivative thermogravimetric) curves of the studied ILs with TFSI anion. Generally, the greater effect on the thermal stability of ILs has the type of anion, then the cation [52]. However, since the tested ILs have the same anion, the influence of the cation on their thermal stability becomes clearly visible. The thermal decomposition of studied ILs was a one-stage process. However, their thermal stability was quite different. Considering the T_5%_, i.e., the decomposition temperature at a 5% mass change, as the onset decomposition temperature, the highest thermal stability was exhibited by the pyrrolidinium ILs (T_5%_ in the range of 418–423 °C), whereas the lowest decomposition temperatures were determined for sulfonium salts (T_5%_ in the range of 278–283 °C). This is in a good agreement with the research results presented by Rennie et al. [24], according to which the decomposition temperature of BMPyrrolTFSI (445 °C) was approximately 150 °C higher than that of TesTFSI (290 °C). Ammonium ILs exhibited T_5%_ in the range of 410–378 °C, so their thermal stability was lower than pyrrolidinium salts, but significantly higher compared to sulfonium ILs. Similar tendency was observed for T_DTG_, so the temperature at which the rate of mass loss is the highest. Therefore, it could be clearly stated that the thermal stability of ILs with TFSI anion strongly depends on their structure, i.e., the type of cation and its substituents. Regarding the influence of cation, very important seems to be the atom on which the positive charge is located. It should be noticed that ILs with positive charge located on the quaternary nitrogen showed significantly higher thermal stability compared to sulfonium salts, in which the positive charge is located on the sulfur atom. On the other hand, the effect of alkyl substituents present in the cation on the thermal stability of ILs depends both on their number and chain length. For pyrrolidinium ILs, increasing the length of alkyl chain from C4 to C8 without changing the number of substituents did not considerably affect the T_5%_ and T_DTG_ temperatures. The differences between the characteristic decomposition temperatures of BMpyrrolTFSI and OMpyrrolTFSI were 5 and 8 °C for T_5%_ and T_DTG_, respectively, and slightly higher thermal stability was shown by the ionic liquid with shorter (butyl) chain. Similar relationship between the thermal stability and chain length of substituents in the cation was observed for ammonium ILs. BmaTFSI, with three methyl groups and one butyl chain on a quaternary nitrogen, exhibited T_5%_ approximately 32 °C higher than MoaTFSI in which two methyl groups and butyl chain were replaced by three long octyl chains. Therefore, it could be stated that thermal stability of ILs with the same anion is improved with shorter chain length of substituents and greater number of short substituents in the cation. The same relationship between the thermal stability of ILs and chain length of the alkyl substituent in the cation was reported by Ngo et al. for alkylammonium ILs with TFSI and bis(pentafluoroethylsulfonyl)imide (BETI) anions [52]. Furthermore, the onset decomposition temperature for BmaTFSI was reported to be 403 °C, which is similar to the results of our study (T_5%_ of approximately 410 °C). Regarding sulfonium ILs, slightly higher T_5%_ (approximately 5 °C) was achieved for DemsTFSI having two ethyl and one methyl group as compared to TesTFSI with three ethyl chains in the sulfonium cation. However, the difference in substituents of sulfonium ILs is very small. These ILs differ in one substituent and its length is only one carbon atom smaller in DemsTFSI in comparison with TesTFSI. A similar behavior was confirmed by Rennie et al. for alkylsulfonium ILs with methyl, ethyl, and propyl substituents in the cation. The thermal stability of the ILs decreased with increasing chain length of the alkyl substituent in the cation. However, these were not very significant differences as the decomposition temperature varied from 295 °C for diethylmethylsulfonium cation to 280 °C for diethylpropylsulfonium cation [24]. Lee et al. also confirmed that the decomposition temperature of ammonium and sulfonium ILs decreased with increasing length of the alkyl substituents in the cation [53]. The effect of chain length on the thermal stability of ILs with different cations is difficult to interpret. A longer chain length results in increased van der Waals forces, which also decrease the intramolecular electrostatic interaction, which leads to an overall reduction in the interaction, and consequently to lower thermal stability. Furthermore, longer alkyl chain could result in both the carbon radicals and carbocation being more stable, which causes the degradation process to proceed more easily [54]. Having known that studied ILs exhibited quite different thermal stability, it is reasonable to investigate their influence on the thermal decomposition of NR vulcanizates. Appropriate results will be presented and discussed later.

### 3.2. Influence of ILs with TFSI Anion on the Vulcanization of NR Compounds and Crosslink Density of Vulcanizates

Cure characteristics such as torque increase (∆S), optimal vulcanization time (t_90_), and scorch time (t_02_) provide information about the impact of ILs and curing system on the rheological properties of NR compounds. Results are presented in Table 4.

Analyzing the minimum rheometric torque (S_min_) values of NR compounds and taking into account the measurement error, it can be concluded that the application of ILs and their structure did not have a significant effect on this parameter. It is worth noting that the minimum torque is a measure of the viscosity of the uncrosslinked rubber compound. Therefore, the incorporation of ILs into the rubber matrix did not alter the viscosity of the uncrosslinked NR compounds. This is important for technological reasons, since the viscosity of uncrosslinked rubber compound significantly affects the processing, especially by extrusion or injection molding. No influence of ILs with the TFSI anion on the S_min_ values was confirmed by Marzec et al. for alkylimidazolium salts with different lengths of alkyl substituents, which were used in the acrylonitrile-butadiene elastomer (NBR) composites [55] and for 1-butyl-3-methylimidazolium bis(trifluoromethylsulfonyl)imide applied in carboxylated acrylonitrile-butadiene elastomer (XNBR) [56]. The reference rubber compound without ionic liquid was characterized by a maximum torque (S_max_) of 8.7 dNm and the torque increase (∆S) of 8.4 dNm, respectively. The application of pyrrolidinium and ammonium ILs increased the S_max_, and consequently ∆S compared to the reference rubber compound, while the sulfonium ILs decreased these parameters to approximately 6 dNm. The increase in torque during rheometric measurement results from the increase in the stiffness of the rubber compound due to the crosslinking process. Hence, the increase in torque directly correlates with the elastomer crosslinking degree (the greater the ΔS, the greater the degree of crosslinking). Therefore, NR compounds with sulfonium ILs are expected to have a lower crosslinking degree than the benchmark and NR compounds with other ILs. Thus, sulfonium ILs could act as retarders of vulcanization. On the other hand, the lowest ΔS of rubber compounds with sulfonium ILs may result from their plasticizing effect. Most importantly, the highest values of ΔS were obtained for NR compounds containing ammonium and pyrrolidinium ILs with longer, i.e., octyl, chain in the cation such as MoaTFSI and OMPyrrolTFSI (∆S of approximately 11 dNm). Thus, the type of cation and the length of alkyl substituent in the cation of ILs affected their activity in the vulcanization. The positive effect of pyrrolidinium ILs with different anions on ΔS and consequently crosslinking degree of the elastomer compounds was reported by Maciejewska et al. [57]. On the other hand, ILs did not have a significant influence on the scorch time t_02_, and thus on the safety of processing NR compounds at 160 °C. The t_02_ of rubber compounds with ILs was in the range of 0.4–0.8 min, so quite similar to that of the benchmark without the ionic liquid (0.7 min). Laskowska et al. presented similar results concerning the influence of TFSI-based ILs with different lengths of substituents in the cation on the scorch time of NBR and XNBR rubber compounds [58,59]. The reference rubber compound exhibited the optimal vulcanization time (t_90_) of approximately 2.5 min. Regardless of the length of alkyl substituent in the cation, pyrrolidinium and ammonium ILs shortened t_90_ by about 1 min, while sulfonium ILs did not considerably affect this parameter as compared with the benchmark. Therefore, application of pyrrolidinium and ammonium ILs with TFSI anion had a positive impact on the cure characteristics—they acted as coagents of vulcanization, whereas sulfonium ILs did not support the vulcanization of NR compounds, acting rather as vulcanization retarders. The positive impact of pyrrolidinium ILs on t_90_ was confirmed by Maciejewska et al. for NBR composites with different fillers [60], whereas Przybyszewska et al. reported that alkylammonium salts shortened t_90_ of unfilled NBR compounds [61].

**Figure 2:**
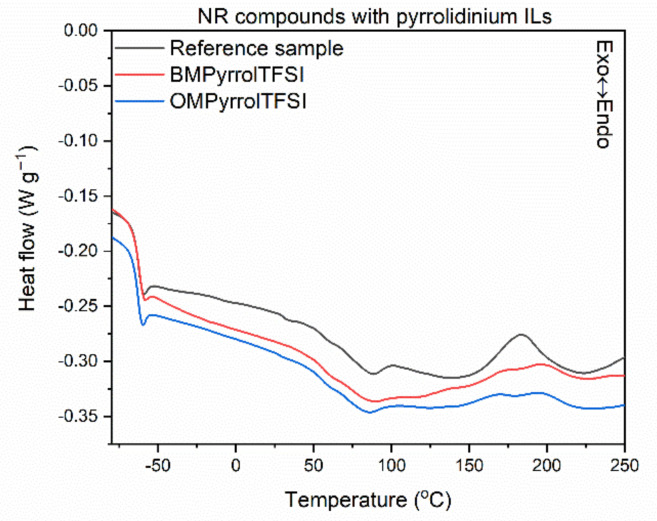
Differential scanning calorimetry (DSC) curves of NR compounds containing pyrrolidinium ILs with TFSI anion.

**Figure 3:**
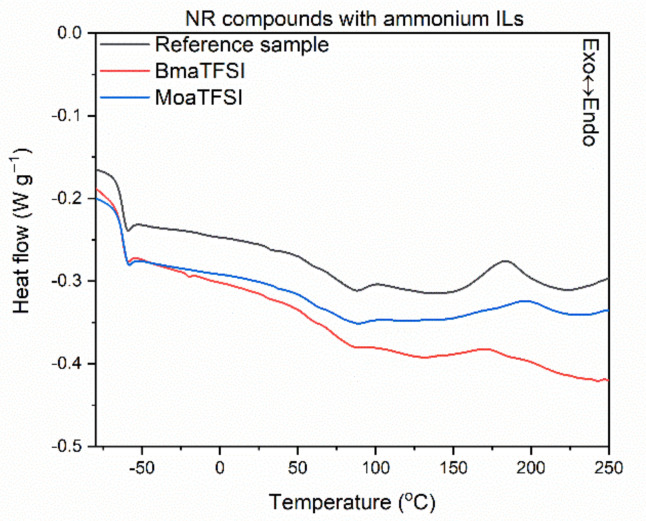
DSC curves of NR compounds containing ammonium ILs with TFSI anion.

**Figure 4:**
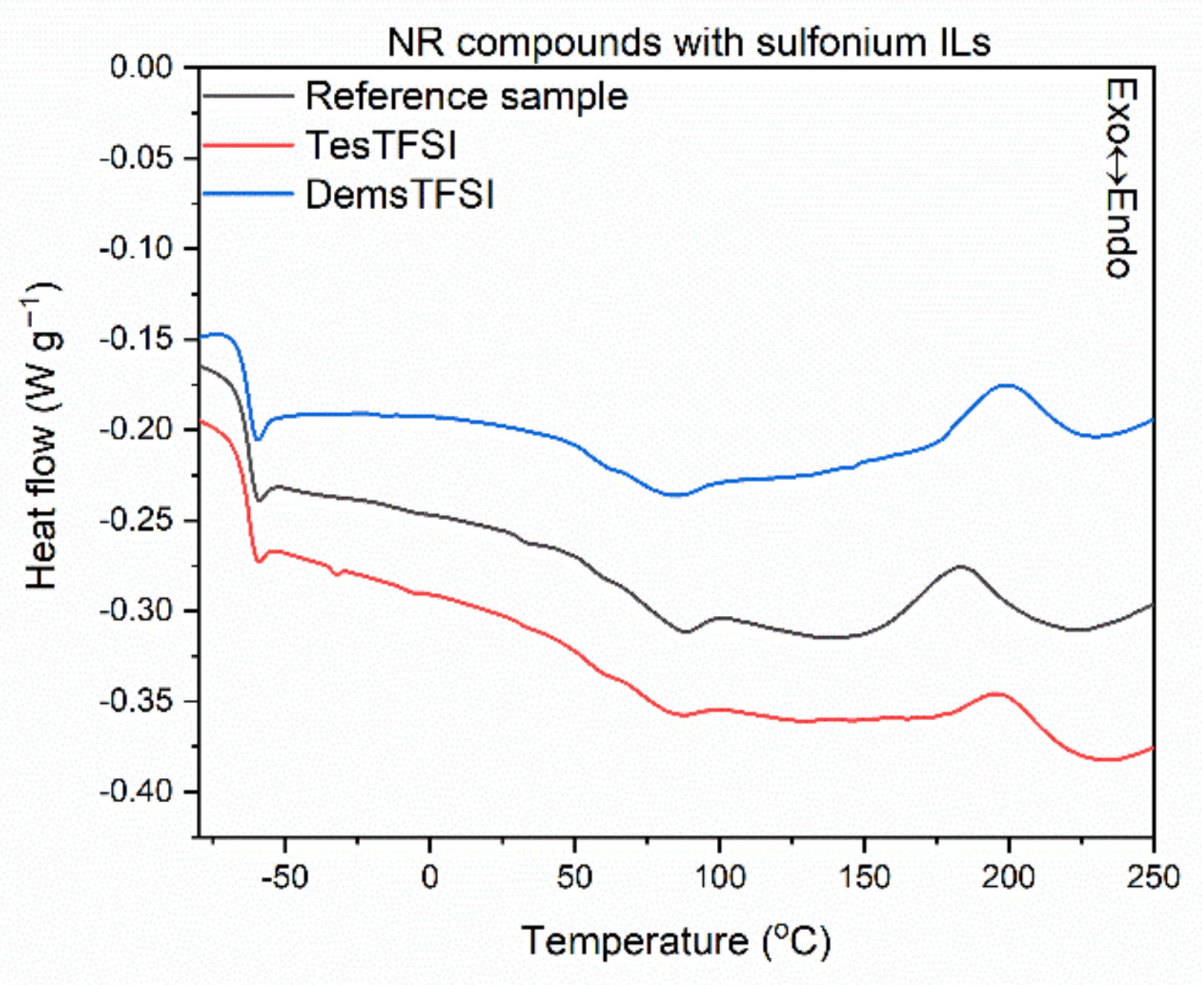
DSC curves of NR compounds containing sulfonium ILs with TFSI anion.

Having investigated the influence of ILs structure on the rheometric properties of NR compounds, we then explored their effect on the temperature and energetic effect (enthalpy) of crosslinking reactions employing DSC analysis. The results for NR compounds are presented in Figure 2, Figure 3 and Figure 4 and Table 5. In addition, the influence of ILs on the glass transition temperature (T_g_) of NR elastomer was examined.

Analyzing the differential scanning calorimetry (DSC) plots, a step on the DSC curves is observed due to the change in the heat capacity (ΔC_p_), which results from the elastomer transition from the glassy state to the elastic region upon heating. A midpoint of this inflection corresponds to the glass transition temperature (T_g_), which is crucial for determining the service temperature range of elastomeric products. Applying ILs with TFSI anion and the structure of their cation did not affect both the T_g_ of the elastomer and the thermal effect of this phase transition, i.e., ΔC_p_. The T_g_ temperature determined for all the vulcanizates tested was approximately −63 °C, so typical for NR elastomer [62]. Crosslinking of the NR compound without ionic liquid proceeded in a temperature range of 148–220 °C with an enthalpy of 7.0 J/g. It was a one-step, exothermic process. The influence of ILs on the crosslinking temperature strongly depends on their structure, and more precisely on the length of the alkyl substituent in the cation. Regarding pyrrolidinium and ammonium ILs, those with butyl substituents in the cation caused a significant reduction in the onset crosslinking temperature (by 16–27 °C) compared to the reference NR compound. Similar effect was achieved for sulfonium salt with three ethyl substituents (TesTFSI). On the other hand, ILs with octyl chains in the cation as well as sulfonium salt DemsTFSI, did not affect the onset crosslinking temperature of NR; the differences were in the range of standard deviation. Reduction in the crosslinking temperature by ionic liquid with BMPyrrol cation was reported by Maciejewska et al. for NBR compounds [57,60]. The same authors confirmed that rubber compounds with alkylimidazolium ILs containing butyl substituents exhibited lower onset crosslinking temperatures than that of rubber compounds with octylimidazolium ILs [63]. Analyzing the DSC curves, the peaks corresponding to crosslinking of NR containing ILs were broader, more asymmetrical, and fuzzier (Figure 2, Figure 3 and Figure 4) than that of the reference rubber compound. The enthalpy of crosslinking was slightly lower for rubber compounds with ILs, especially pyrrolidinium and ammonium salts with butyl chains, as compared to the benchmark without IL. This may suggest a lower intensity or efficiency of crosslinking, but this conclusion should not be drawn without the analysis of the crosslink density of the vulcanizates.

Therefore, in the next step of the studies, the effect of ILs and their structure on the crosslink density of NR vulcanizates was determined using the equilibrium swelling method and the results are presented in Table 6.

It is commonly known that the elastomeric network formed by vulcanization restricts the absorption of the solvent by the vulcanizate. Thus, the equilibrium swelling is inversely proportional to the crosslink density of the vulcanizate. The reference vulcanizate without ionic liquid exhibited the crosslink density of 1.40 × 10^−5^ mole/cm^3^. As it was expected after analysis of rheometric tests results, vulcanizates with pyrrolidinium and ammonium ILs showed higher crosslink density, and consequently lower equilibrium swelling in toluene, than the benchmark without IL. Most importantly, following the results of rheometric tests, the highest crosslink densities were obtained for NR vulcanizates containing ILs with longer, i.e., octyl, substituent in the cation such as OMPyrrolTFSI and MoaTFSI as compared to salts with butyl chains. Thus, the results of the equilibrium swelling measurements also confirmed that the type of cation and the length of alkyl substituent in the cation of ILs affected their activity in the vulcanization. Accordingly, Przybyszewska et al. [61] found that the crosslink density of vulcanizates containing some alkylimidazolium ILs increased with increasing length of alkyl chains attached to the imidazolium ring. Vulcanizates with sulfonium ILs exhibited a crosslink density lower than the reference sample, which also correlates with the results of rheometric tests. However, the structure of sulfonium cation did not significantly affect the crosslink density of the vulcanizates; the differences were in the range of experimental error. Most importantly, ILs with TFSI anion and pyrrolidinium or ammonium cations can be used to improve the crosslink density of the NR vulcanizates filled with CB. Similar results were achieved for NBR vulcanizates with alkylpyrrolidinium ILs [57,60] and alkylammonium bromides [61]. Moreover, Yasin et al. reported the positive effect of the ionic liquid, i.e., 1-ethyl-3-methylimidazolium acetate on the crosslink density of NR composites [40].

### 3.3. The Effect of ILs with TFSI Anion on Mechanical Performance of NR Vulcanizates

To establish the influence of the ILs with TFSI anion on the mechanical properties of NR vulcanizates under static conditions, their behavior during stretching at a constant speed was investigated. Moreover, the effect of ILs on the hardness of the vulcanizates was explored. The results are presented in Table 7.

The stress at a relative elongation of 300% (SE_300_ modulus) correlates with the crosslink density of the vulcanizate; the higher the crosslink density, the greater the SE_300_ modulus of the vulcanizate. Pyrrolidinium and ammonium ILs with butyl chain, such as BMPyrrolTFSI and BmaTFSI, did not have a considerable influence on SE_300_, while ILs with octyl substituent caused an increase in SE_300_ as compared to the reference vulcanizate without the ionic liquid. This resulted from the higher crosslink density of the vulcanizates containing ILs with octyl chains in the cation. On the other hand, the sulfonium ILs reduced the SE_300_ by approximately 1 MPa compared to the benchmark due to the lower crosslink density of the vulcanizates.

Similarly to SE_300_, the structure of the ILs had an impact on the tensile strength (TS) of the NR vulcanizates, which resulted from the influence of particular ILs on the crosslink density. The TS of the reference vulcanizate without IL was 19.5 MPa. Application of pyrrolidinium and ammonium ILs with octyl chain in the cation enhanced the TS by approximately 1.6–3.6 MPa, whereas ILs with butyl substituent did not considerably affect this property compared to the benchmark (the differences were within the range of standard deviation). On the other hand, owing to the lower crosslink density of the vulcanizates, sulfonium ILs deteriorated the TS by more than 4 MPa as compared to the reference sample without IL. The beneficial influence of TFSI-based ILs on the TS of elastomers was reported for different alkylimidazolium salts [58,59]. On the other hand, alkylpyrrolidinium ILs with different cations improved the tensile properties of NBR vulcanizates, whereas alkylimidazolium ILs, especially those with 1-hexyl-3-methylimidazolium cation, were found to improve the TS of NR vulcanizates with multiwalled carbon nanotubes [37].

Despite changes in the crosslink density of the vulcanizates, ILs with the TFSI anion did not have a significant effect on their flexibility. Reference vulcanizate exhibited an elongation at break of 572%, whereas EB of the ILs-containing elastomers was in the range of 518–586%. The lowest EB was demonstrated by the vulcanizates with the highest crosslink density, i.e., those containing ILs with octyl chains in the cation (518% and 542% for the vulcanizate with OMpyrrolTFSI and MoaTFSI, respectively).

Most of ILs tested did not significantly alter the hardness of the NR vulcanizates as compared with the benchmark, which exhibited the hardness of 43 ShA. ILs-containing vulcanizates showed the hardness in the range of 39–48 ShA. As it was expected, the lowest hardness was determined for the vulcanizates with sulfonium salts due to their lower crosslink density.

Most importantly, application of pyrrolidinium and ammonium ILs with TFSI anion allowed to obtain NR vulcanizates with improved tensile strength, without significantly affecting their flexibility and hardness compared to the benchmark without IL. On the other hand, when a lower hardness of the material is required for a particular application without its very high tensile strength, sulfonium ILs can be used and thus, the desired properties can be provided.

### 3.4. The Effect of ILs with TFSI Anion on Thermo-Oxidative Aging of NR Vulcanizates

**Figure 5:**
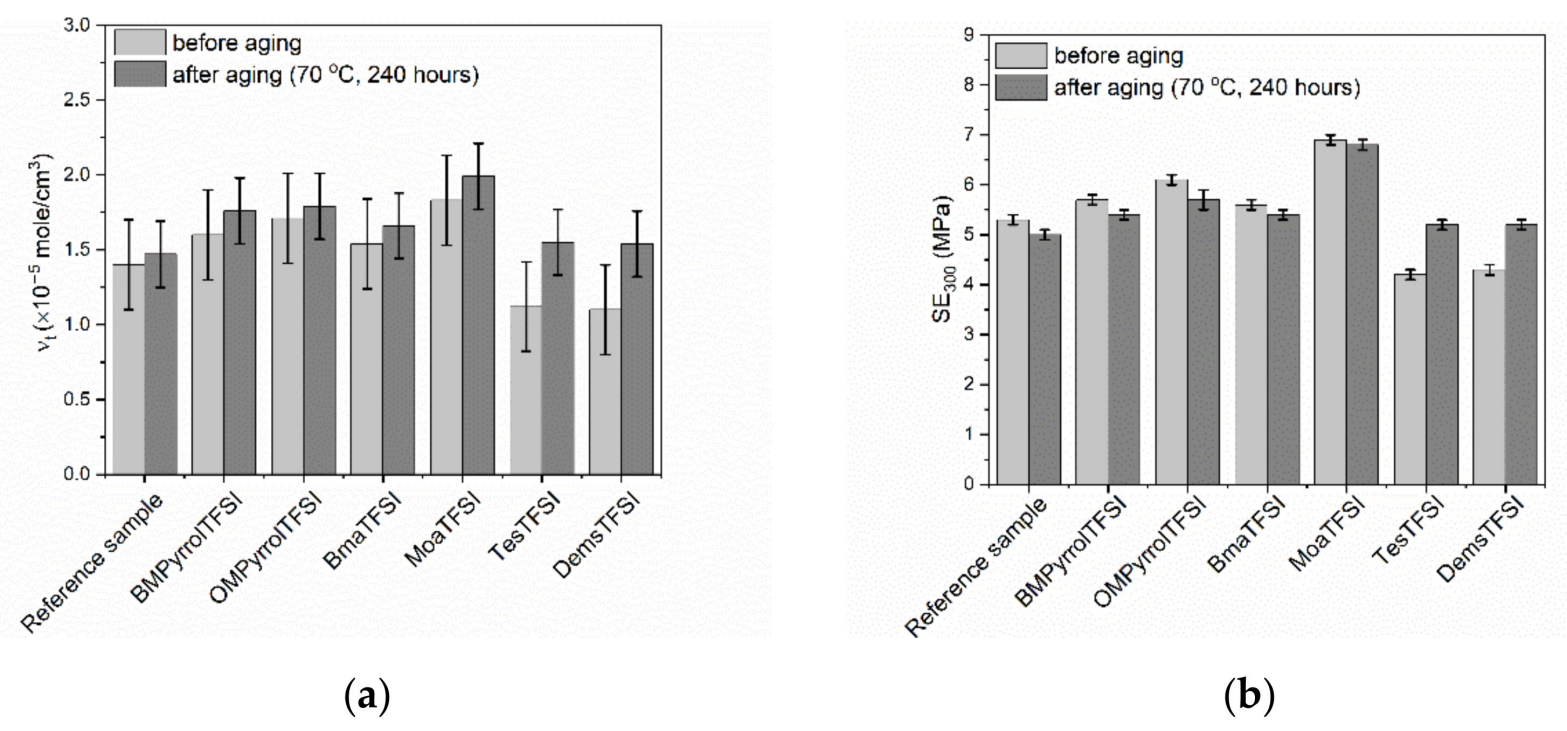

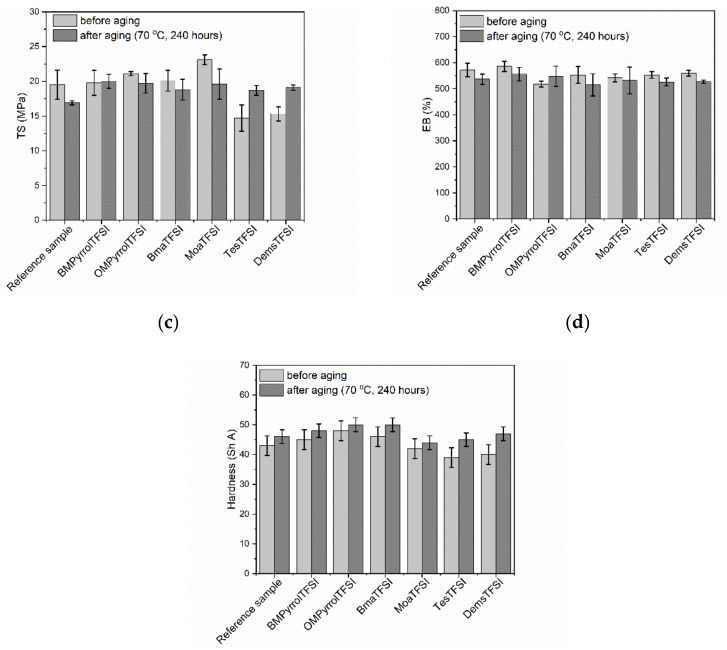
Influence of thermo-oxidative aging on the properties of NR vulcanizates containing ILs with TFSI anion: (**a**) crosslink density; (**b**) stress at 300% relative elongation; (**c**) tensile strength; (**d**) elongation at break; (**e**) hardness.

One of the few weaknesses of NR is its poor aging resistance compared to most synthetic rubbers. Therefore, if ILs are to be used to improve the crosslinking characteristics and/or performance of the NR composites, they should not deteriorate the aging resistance. Consequently, the influence of ILs with TFSI anion on the resistance of NR vulcanizates to thermo-oxidative aging was investigated. NR vulcanizates were stored at 70 °C for 240 h, and then their crosslink density, tensile properties, and hardness were tested and compared with the same properties of non-aged vulcanizates. The impact of prolonged thermo-oxidation on the performance of NR vulcanizates is presented in Figure 5.

As expected, the thermo-oxidative aging increased the crosslink density of both the benchmark and the vulcanizates with ILs (Figure 5a). The highest increase in the crosslink density after thermo-oxidation was observed for the vulcanizates with sulfonium ILs, which were characterized with the lowest crosslink densities before aging process. It probably resulted from the fact that ILs with sulfonium cation acted as vulcanization retarders, which reduced the efficiency of crosslinking system consumption during vulcanization. Consequently, some unreacted crosslinkers remained inside the elastomer matrix and prolonged exposure to elevated temperature during thermo-oxidative aging could initiate further crosslinking of the elastomer. Since SE_300_ depends on the crosslink density, vulcanizates with sulfonium ILs exhibited significantly higher SE_300_ after thermo-oxidative aging as compared to the non-aged samples (Figure 5b). Other vulcanizates demonstrated only slight changes in SE_300_ upon thermo-oxidation. Thermo-oxidative aging reduced the tensile strength of the reference vulcanizate by approximately 3 MPa (Figure 5c). On the other hand, prolonged thermo-oxidation did not significantly affect the TS of vulcanizates with pyrrolidinium ILs and BmaTFSI, whereas TS of the vulcanizate with MoaTFSI was reduced by approximately 3.5 MPa compared to the non-aged vulcanizates. It should be noticed that MoaTFSI-containing vulcanizate showed significant increase in the crosslink density resulting from aging process. However, this vulcanizate was also characterized by the highest crosslink density before the thermo-oxidative aging. Thus, further crosslinking during the aging process could cause this elastomer to be over-crosslinked, and consequently brittle and less resistant to mechanical stress. It is commonly known that the tensile strength increases with the crosslink density of the vulcanizate to a certain critical value of the crosslink density, above which the vulcanizate becomes over-crosslinked. Afurther increase in the crosslink density of the vulcanizate results in a deterioration of the tensile strength [64].

Most importantly, the opposite influence of thermo-oxidative aging on the TS was observed for the vulcanizates with sulfonium ILs. Their tensile strength improved by approximately 4 MPa due to increase in the crosslink density of these vulcanizates after thermo-oxidative aging. Since the vulcanizates with sulfonium ILs exhibited significantly lower crosslink density before the thermo-oxidative aging compared to the benchmark or vulcanizates with other ILs, formation of additional crosslinks in the elastomer network during prolonged exposure to elevated temperature did not result in the over-crosslinking. Thermo-oxidative aging did not significantly affect the flexibility of NR vulcanizates. Most of vulcanizates were characterized by slightly lower EBs (Figure 5d) compared to non-aged samples due to their higher crosslink density. The hardness of all vulcanizates after thermo-oxidative aging was higher than before this process, and it was in the range of 44–50 ShA (Figure 5e). As expected, the highest increase in the hardness after thermo-oxidative aging (by approximately 7 ShA) was observed for the vulcanizates containing sulfonium ILs, that correlates with the highest increase in the crosslink density.

To facilitate the assessment of the influence of ILs with TFSI anion on the resistance of vulcanizates to thermo-oxidative aging, the aging factor AF was calculated based on the changes in the tensile properties (TS and EB) of vulcanizates due to the aging process (Table 8).

It is known that NR rubber composites have poor resistance to accelerated aging processes [34]. Reference vulcanizate without ionic liquid was characterized by AF of approximately 0.8, so it exhibited quite good resistance to thermo-oxidative aging. The type of ILs cation had a significant impact on the resistance of NR to prolonged thermo-oxidation. Ammonium ILs and their structure did not significantly affect the resistance of NR vulcanizates to thermo-oxidation compared to the reference vulcanizate. Pyrrolidinium ILs improved the aging resistance of NR vulcanizates, increasing the AF value to approximately 1. The same effect was achieved for sulfonium ILs, which increased the AF value to approximately 1.2, so significantly enhanced the resistance of NR vulcanizates to thermo-oxidative aging due to the improvement of their tensile strength resulting from the aging process. Most importantly, depending on the cation type, ILs with TFSI anion can be used to fine-tune the resistance of NR vulcanizates to thermo-oxidative aging. The beneficial impact of ILs with variety of cations and anions on the resistance to thermo-oxidative aging was previously confirmed by Maciejewska et al. for different elastomers, such as butadiene-styrene (alkylammonium and benzalkonium ILs) or hydrogenated acrylonitrile-butadiene elastomer (alkylimidazolium ILs) [65,66].

### 3.5. The Effect of ILs with TFSI Anion on Thermal Stability of NR Vulcanizates

**Figure 6:**
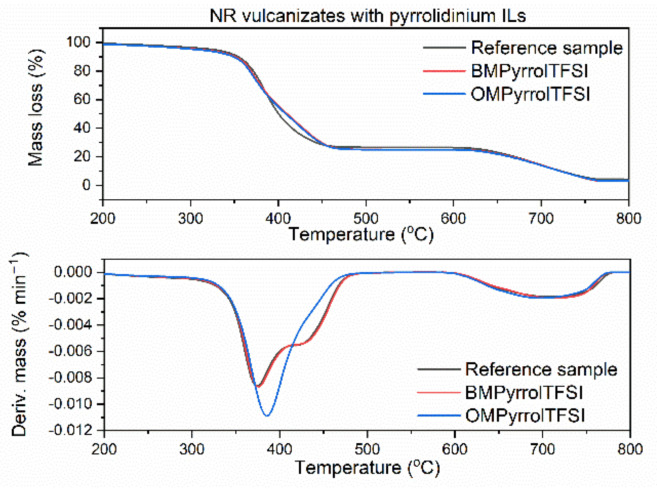
Thermogravimetric (TG) and derivative thermogravimetric (DTG) curves of the NR vulcanizates containing pyrrolidinium ILs with TFSI anion.

**Figure 7:**
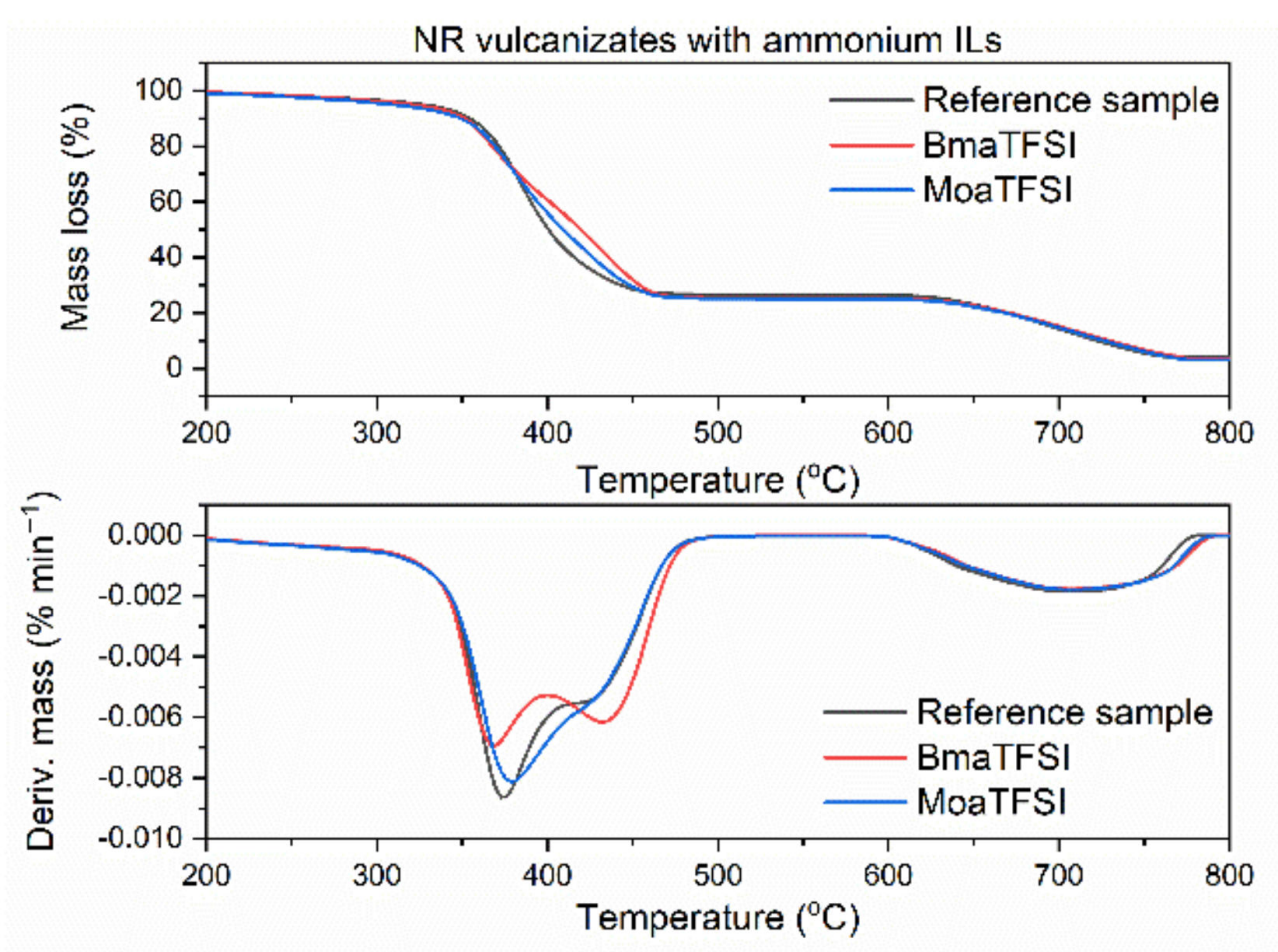
TG and DTG curves of the NR vulcanizates containing ammonium ILs with TFSI anion.

**Figure 8:**
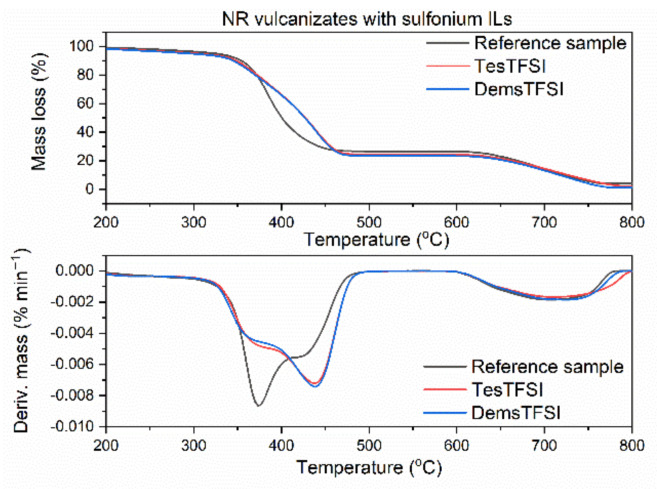
TG and DTG curves of the NR vulcanizates containing sulfonium ILs with TFSI anion.

TG analysis revealed that pure ILs with TFSI anion exhibited different thermal stability, which was strongly dependent on the structure of their cation. Thermal stability of elastomer composites results from thermal behavior of both elastomer matrix and components of rubber compounds. Therefore, the influence of ILs and their structure on the thermal stability of NR vulcanizates was examined and the results are shown in Table 9 and in Figure 6, Figure 7 and Figure 8.

TG measurements were performed in two stages. An inert gas—argon, was used in the first stage (25–600 °C), which caused the pyrolysis of elastomer and organic ingredients such as 2-mercaptobenzothiazole (MBT), stearin, and ILs. Therefore, the mass losses in the temperature range of 25–600 °C of the vulcanizates containing ILs were slightly higher than for the reference vulcanizate (Table 9). Above 600 °C argon was replaced by air and the measurement was continued to 900 °C, so the mass loss in the temperature range of 600–900 °C corresponds to the combustion of carbon black (filler) and the residues after thermal decomposition. Due to the same content of filler the mass loss in this range of temperature was similar for all vulcanizates (approximately 22%). The mineral residue after decomposition was in the range of 1.8–4.3% and resulted from the content of zinc oxide in the vulcanizates, and from the ash remaining after combustion of carbon black and the residues after thermal decomposition.

Reference vulcanizate without ionic liquid began to thermally decompose at the temperature approximately 325 °C. Pyrrolidinium ILs slightly deteriorated thermal stability of the vulcanizates (Figure 6). The T_5%_ temperature decreased by 12 °C for the vulcanizate containing the IL with butyl chain (BMPyrrolTFSI) and by 18 °C for the IL with octyl chain in the cation (OMPyrrolTFSI). This may result from the lower thermal stability of pure OMPyrrolTFSI (T_5%_ 418 °C) as compared to pure BMPyrrolTFSI (T_5%_ 423 °C). Similar effect of ILs with TFSI anion on the thermal stability was reported by Laskowska et al. for XNBR elastomer. Moreover, the T_5%_ of the XNBR vulcanizates decreased with increasing length of the alkyl substituent in the ionic liquid cation [59]. Regarding the ammonium ILs (Figure 7), BmaTFSI slightly affected the onset decomposition temperature of NR reducing T_5%_ by 3 °C compared to the reference vulcanizate. On the other hand, T_5%_ of the vulcanizate with MoaTFSI was 18 °C lower than that of the benchmark, which correlates well with the significantly lower thermal stability of pure MoaTFSI compared to BmaTFSI. Reduction in T_5%_ temperature by long-chain alkylammonium ILs with different anions was also reported for styrene-butadiene elastomer [65]. The poorest thermal stability (T_5%_ of approximately 300 °C) was demonstrated by the vulcanizates with sulfonium ILs (Figure 8) and resulted from the lowest decomposition temperatures of pure ILs with sulfonium cation (T_5%_ of approximately 278 °C and 283°C for TesTFSI and DemsTFSI, respectively). It should be noted that despite sulfonium ILs lowered the onset decomposition temperature of NR vulcanizates, they reduced the rate of thermal decomposition compared to the benchmark and vulcanizates with other ILs. NR vulcanizates with sulfonium ILs exhibited T_DTG_ temperature of 456 °C, whereas for other vulcanizates, T_DTG_ was in the range of 386–397 °C. Thus, decomposition of the vulcanizates with sulfonium ILs began at lower temperatures but proceeded slower as compared with other tested samples. Despite ILs with TFSI anion reduced the thermal stability of NR, the vulcanizates were thermally stable up to a temperature of approximately 300 °C, which is sufficient for their potential application in technology.

## 4. Conclusions

The possibility of application of ILs with different cations and TFSI anion for fine-tuning the cure characteristics and performance of elastomer composites based on a biodegradable natural rubber (NR) matrix was investigated.

The structure of ILs cation, i.e., pyrrolidinium, ammonium, sulfonium, and the length of alkyl substituents in the cation significantly affected the activity of ILs in the crosslinking of NR elastomer, and consequently the cure characteristics of rubber compounds, crosslinks density of the vulcanizates, as well as their mechanical properties and resistance to thermo-oxidative aging. As expected, the type of cation and the length of substituents had an essential influence on the thermal stability of pure ILs; those with pyrrolidinium and ammonium cation (positive charge located on nitrogen atom) exhibited much better thermal stability than ILs with sulfonium cation. No less important was the effect of alkyl chains length in the cation. Increasing the length of alkyl substituents in the cation from C4 to C8 enhanced the temperature of ILs decomposition. Thermal stability of ILs had a meaningful influence on the thermal decomposition temperature of NR vulcanizates. It should be noticed that thermal stability of the vulcanizates fully correlated with the thermal stability of pure ILs. Although ILs reduced the temperature of NR decomposition (especially those with sulfonium cation), the vulcanizates were thermally stable to a temperature of approximately 300 °C.

ILs with TFSI anion slightly reduced the optimal vulcanization time, while no considerable effect on the scorch time was observed, which is crucial for the safe processing of NR compounds at elevated temperatures. Moreover, the range of vulcanization temperature was slightly reduced by using ILs with TFSI anion as compared to the reference compound without ionic liquid. Pyrrolidinium and ammonium ILs, especially those with octyl substituents, enhanced the torque increase during vulcanization of NR compounds due to the higher crosslinking degree of the elastomer. Hence, pyrrolidinium and ammonium ILs can be used to support the vulcanization of NR compounds. In the case of sulfonium ILs, a reduction in torque increase was observed resulting from the lower crosslinking degree of NR as compared to the benchmark without IL. Thus, sulfonium ILs are supposed to act as vulcanization retarders, diminishing the efficiency of vulcanization. Most importantly, performed studies revealed the ILs with TFSI anion can be successfully used to fine-tune the cure characteristics and crosslinking degree of NR compounds.

Reduction in the crosslink density caused by sulfonium ILs resulted in the lower tensile strength and slightly lower hardness of the vulcanizates in comparison with the reference sample, but similar flexibility of the material. On the other hand, pyrrolidinium and ammonium ILs improved mechanical properties of the vulcanizates. Moreover, pyrrolidinium and sulfonium ILs with TFSI anion enhanced the resistance of NR vulcanizates to thermo-oxidative aging, which is important for technological applications.

## Figures and Tables

**Table 1:** Ionic liquids (ILs) with TFSI anion used in natural rubber (NR) compounds.

Name	Abbreviation	CAS Number	Purity (%)	Water Content (wt %)
**Pyrrolidinium ILs**				
1-butyl-1-methylpyrrolidinium bis (trifluoromethylsulfonyl)imide ^1^	BMPyrrolTFSI	223437-11-4	≥99.0	≤0.01
1-methyl-1-octylpyrrolidinium bis(trifluoromethylsulfonyl)imide ^2^	OMPyrrolTFSI	927021-43-0	>99.0	<0.01
**Ammonium ILs**				
N-butyl-N-trimethylammonium bis(trifluoromethylsulfonyl)imide ^1^	BmaTFSI	258273-75-5	≥98.5	≤0.2
Methyltrioctylammonium bis (trifluoromethylsulfonyl)imide ^1^	MoaTFSI	375395-33-8	≥99.0	≤0.03
**Sulfonium ILs**				
Triethylsulfonium bis(trifluoromethylsulfonyl)imide ^1^	TesTFSI	321746-49-0	≥95.0	≤0.02
Diethylmethylsulfonium bis(trifluoromethylsulfonyl)imide ^2^	DemsTFSI	792188-85-3	>99.0	<0.01

^1^—ILs provided by Sigma-Aldrich, Poznań, Poland; ^2^—ILs manufactured by IoLiTec Ionic Liquids Technologies GmbH, Heilbronn, Germany.

**Table 2:** General formula of the NR compounds, parts per hundred of rubber (phr); MBT, 2-mercaptobenzothizole, CB, carbon black.

Ingredient	Reference Sample	NR Compounds with ILs
NR	100	100
MBT	2	2
Sulfur	2	2
ZnO	5	5
Stearin	1	1
CB	30	30
Ionic liquid ^1^	-	3

^1^ Applied ionic liquids are listed in Table 1.

**Table 3:** Thermal stability of ILs with TFSI anion (T_5%_, decomposition temperature at 5% of the mass change; T_DTG_, DTG peak temperature; standard deviations (SD): T_5%_, T_DTG_ ± 3 °C).

Ionic Liquid	T_5%_ (°C)	T_DTG_ (°C)
Pyrrolidinium ILs		
BMPyrrolTFSI	423	480
OMPyrrolTFSI	418	472
Ammonium ILs		
BmaTFSI	410	485
MoaTFSI	378	442
Sulfonium ILs		
TesTFSI	278	333
DemsTFSI	283	343

**Table 4:** Cure characteristics at 160 °C for NR compounds containing ILs with TFSI anion (S_min_, minimum torque; S_max_, maximum torque; ∆S, torque increase; t_02_, scorch time; t_90_, optimal vulcanization time; SD: S_min_ ± 0.1 MPa; S_max_ ± 2.0 MPa; ∆S ± 2.0 MPa; t_02_ ± 0.1 min.; t_90_ ± 0.3 min.).

NR Compounds	S_min_ (dNm)	S_max_ (dNm)	∆S (dNm)	t_02_ (min)	t_90_ (min)
Reference sample	0.3	8.7	8.4	0.7	2.5
Pyrrolidinium ILs					
BMPyrrolTFSI	0.2	9.8	9.6	0.5	1.8
OMPyrrolTFSI	0.2	10.6	10.4	0.4	1.7
Ammonium ILs					
BmaTFSI	0.2	9.7	9.5	0.5	1.8
MoaTFSI	0.2	11.2	11.0	0.5	1.6
Sulfonium ILs					
TesTFSI	0.2	6.3	6.1	0.7	2.2
DemsTFSI	0.1	5.8	5.7	0.8	2.3

**Table 5:** Temperature and enthalpy of crosslinking reactions measured by differential scanning calorimetry (DSC) for NR compounds containing ILs with TFSI anion (∆H, enthalpy of crosslinking; T_g_, glass transition temperature; ∆C_p,_ heat capacity; SD: temperature ± 9.0 °C; ΔH ± 2.6 J/g; T_g_ ± 1 °C; ∆C_p_ ± 0.1 J/g × K).

NR Compounds	Temperature of Crosslinking (°C)	∆H (J/g)	T_g_ (°C)	∆C_p_ (J/g × K)
Reference sample	148–220	7.0	−62.7	0.41
Pyrrolidinium ILs				
BMPyrrolTFSI	121–225	5.6	−62.5	0.43
OMPyrrolTFSI	145–219	3.4	−63.0	0.42
Ammonium ILs				
BmaTFSI	132–219	5.2	−62.6	0.42
MoaTFSI	149–226	4.1	−62.2	0.42
Sulfonium ILs				
TesTFSI	134–224	6.1	−62.9	0.42
DemsTFSI	146–228	6.8	−63.0	0.40

**Table 6:** Equilibrium swelling in toluene (Q_t_) and crosslink density (ν_t_) of NR vulcanizates containing ILs with TFSI anion (SD: Q_t_ ± 0.4; ν_t_ × 10^−5^ ± 0.3 mole/cm^3^).

NR Vulcanizates	Q_t_ (-)	$\nu t_{\;}\times \;10^{-5}\;$ (mole/cm^3^)
Reference sample	3.67	1.40
Pyrrolidinium ILs		
BMPyrrolTFSI	3.44	1.60
OMPyrrolTFSI	3.32	1.71
Ammonium ILs		
BmaTFSI	3.50	1.54
MoaTFSI	3.20	1.83
Sulfonium ILs		
TesTFSI	4.16	1.12
DemsTFSI	4.20	1.10

**Table 7:** Tensile properties and hardness of NR vulcanizates containing ILs with TFSI anion (SE_300_, stress at 300% relative elongation; TS, tensile strength; EB, elongation at break; H, hardness; SD: SE_300_ ± 1 MPa; TS ± 0.7 MPa; EB ± 8%; H ± 3 ShA).

NR Vulcanizates	SE_300_ (MPa)	TS (MPa)	EB (%)	H (ShA)
Reference sample	5.3	19.5	572	43
Pyrrolidinium ILs				
BMPyrrolTFSI	5.7	19.8	586	45
OMPyrrolTFSI	6.1	21.1	518	48
Ammonium ILs				
BmaTFSI	5.6	20.1	553	46
MoaTFSI	6.9	23.1	542	42
Sulfonium ILs				
TesTFSI	4.2	14.7	553	39
DemsTFSI	4.3	15.3	560	40

**Table 8:** Thermo-oxidative aging factor (AF) of NR vulcanizates (SD: AF ± 0.1).

Vulcanizate	AF (-)
Reference sample	0.8
Pyrrolidinium ILs	
BMPyrrolTFSI	1.0
OMPyrrolTFSI	1.0
Ammonium ILs	
BmaTFSI	0.9
MoaTFSI	0.8
Sulfonium ILs	
TesTFSI	1.2
DemsTFSI	1.2

**Table 9:** Onset temperature of thermal decomposition (T_5%_), DTG peak temperature (T_DTG_), and total mass loss (∆m) during decomposition of NR vulcanizates (SD: T_5%_ ± 1.3 °C; T_DTG_ ± 1.2°C; ∆m ± 1.3%).

Vulcanizate	T_5%_ (°C)	T_DTG_ (°C)	Δm_(25–600 °C)_ (%)	Δm_(600–900 °C)_ (%)	Residue at 900 °C (%)
Reference sample	325	403	73.4	22.3	4.3
Pyrrolidinium ILs					
BMPyrrolTFSI	313	393	74.3	21.8	3.9
OMPyrrolTFSI	307	392	74.4	21.9	3.7
Ammonium ILs					
BmaTFSI	322	386	74.7	21.8	3.5
MoaTFSI	307	397	74.7	22.0	3.3
Sulfonium ILs					
TesTFSI	304	456	75.2	22.1	2.7
DemsTFSI	295	456	76.0	22.2	1.8

## Data Availability

The data presented in this study are available on request from the corresponding author.
